# Determination of the predictive factors of long-lasting insecticide-treated net ownership and utilisation in the Bamenda Health District of Cameroon

**DOI:** 10.1186/s12889-017-4155-5

**Published:** 2017-03-16

**Authors:** Eric B. Fokam, Germaine F. Kindzeka, Leonard Ngimuh, Kevin T. J. Dzi, Samuel Wanji

**Affiliations:** 1 Department of Zoology and Animal Physiology, University of Buea, PO Box 63, Buea, Cameroon; 2 Epidemiology and Control of Infectious Diseases, Department of Microbiology and Parasitology, University of Buea, PO Box 63, Buea, Cameroon; 3 Research Foundation in Tropical Diseases and Environment, PO Box 474, Buea, Cameroon

## Abstract

**Background:**

Malaria is a serious health concern in Africa. In Cameroon, an endemic country where malaria remains a major public health problem, several control measures have been put in place among which the use of insecticide-treated bednets (LLINs/ITNs) is considered one of the core vector control strategies. However, the greatest challenges include ownership and utilisation by individuals and households. Factors such as age, marital status, gender, education and occupation of the household head, household size, knowledge of bednets, socioeconomic status, and environmental factors have been suggested to have an impact on bednet ownership and utilisation in different settings. The present study sought to determine bednet ownership and utilisation rates and to assess the impact of predictive factors on bednet ownership and use in the Bamenda Health District (BHD) of Cameroon.

**Methods:**

A cross-sectional study involving 384 households was conducted in six health areas in the BHD. A structured and semi-structured questionnaire was used to collect data on demographic and household characteristics as well as information on their bednet ownership and utilisation. Descriptive statistics, bivariate and multivariate logistic regression analysis were performed.

**Results:**

Frequency of bednet ownership was relatively high (63.5%) with LLINs being most abundant (91.9%); the majority of households (87.7%) obtained their bednets during the 2011 free distribution campaign. Utilisation was relatively high (69.3%), with negligence (29.3%) and heat discomfort (26.7%) accounting most for non-usage of bednets. Children less than 5 years (63%) and pregnant women (60%) most often used these nets. Households headed by a married couple, those with older household heads, household with smaller size (5–12 persons), and knowledge of bednets (good knowledge) had positive impacts on bednet ownership (*p* < 0.05). The gender of the household head (males), their educational level, environmental conditions (presence of suitable mosquito breeding sites), bednet number in households (greater number of bednets) and the prioritised groups (children < 5 and pregnant women) had positive impacts on bednet utilisation in households (*p* < 0.05). There was a negative association between bednet ownership and utilisation by households as bednet ownership was high and utilisation of these nets was low. Marital status and age of household head, household size, and knowledge of bednets had impacts on bednet ownership while gender and educational level of the household head, environmental suitability, the number of bednets and the two prioritised groups had an impact on bednet usage.

**Conclusion:**

These factors may be relevant for policy makers and in decision making for the intensification of campaign strategies to ensure more effective subsequent distribution campaigns in the BHD and beyond.

**Electronic supplementary material:**

The online version of this article (doi:10.1186/s12889-017-4155-5) contains supplementary material, which is available to authorized users.

## Background

Malaria infection has constituted a major global health challenge for decades, globally putting an estimated 3.4 billion people at risk, with 214 million cases occurring in 2015 and 438,000 deaths [1]. Most cases of disease (80%) and death (90%) occurred in sub-Saharan Africa, and most fatalities (77%) were in children under 5 years of age. *Plasmodium falciparum* and *P. vivax* are the most important pathogens, the former being the most deadly form and predominates in Africa. Pregnant women and children under the age of five are the most vulnerable groups. The WHO estimates that every 50 s a child dies of malaria, thus it is a major public health problem and an impediment to economic development.

In Cameroon an endemic country, malaria remains a major public health problem as the entire population of over 20 million inhabitants is at risk, with 15 million people at a higher risk [1]. The estimated number of reported cases in 2013 stood between 3,400,000–7,500,000 and malaria accounted for 18% of deaths occurring in health facilities in the country [2]. Several measures for the control of malaria have been put in place. These include the use of chemotherapeutic agents and vector control strategies. Measures related to chemotherapy are: management of confirmed cases with quality Artemisinin-based Combination Therapy (ACTs) and Intermittent Preventive Treatment (IPT) with Sulphadoxine-Pyrimethamine (SP) for pregnant women. Measures related to vector control include the promotion and distribution of long-lasting insecticide-treated nets (LLINs) and environmental hygiene to reduce mosquito breeding sites. Since the adoption in Cameroon of insecticide treated nets (ITNs) as a key preventive tool in 2002, several free distribution campaigns of ITNs were carried out with an objective to have 80% of children below 5 years sleeping under LLINs by 2015. While 52% of households possessed a bednet, only 36% owned a conventionally treated ITN; only 28% of children below 5 years of age were reported to have slept under a net, with 21% under a treated net [2]. Thus to increase bednet possession, the Ministry of Public Health through the National Malaria Control Programme (NMCP) in 2011, launched a national campaign for mass-distribution of LLINs free of charge to households to achieve universal coverage.

Long-lasting insecticide-treated bednets/insecticide-treated bednets (LLINs/ITNs) are considered one of the major components of the selective vector control strategies [3]. However, the greatest challenges include bednet ownership, their effective use by individuals, and replacement of old and torn nets [4]. These challenges prompt the need for evaluation of bednet ownership and proper use to secure the long-term benefits of this control method. Although numerous studies have suggested that several factors influence net ownership and use at the individual level (knowledge of bednets, age, gender, marital status, educational level and occupation of the household head) [5–8] and household factors (including: distance to nearest health service, accessibility to transport, household size, bednet density and the household socioeconomic status) as found in the Malaria Indicator Survey (MIS) data collection tools (http://Malariasurveys.org/surveys.cfm). There is limited information about the population demographics and their association to bednet coverage in Cameroon in general, and in the Bamenda Health District (BHD) specifically. Amidst a second nationwide mass-campaign of free distribution of LLINs by the government through the Ministry of Public Health and National Malaria Control Committee, the situation of bednet ownership and usage remains worrying; this could be attributed to individual and household factors affecting ownership and proper utilisation. From WHO recommendations for household surveys, it is therefore important to investigate the impact of predictive factors of bednet ownership and usage in order to eventually improve bednet possession, acceptance and use in the fight against malaria. This study sought to determine bednet coverage and predictive factors that affect effective ownership and utilisation of bednets. This in turn would have a major bearing on planning for success in ongoing and subsequent distribution efforts.

## Methods

### Study area

**Fig. 1:**
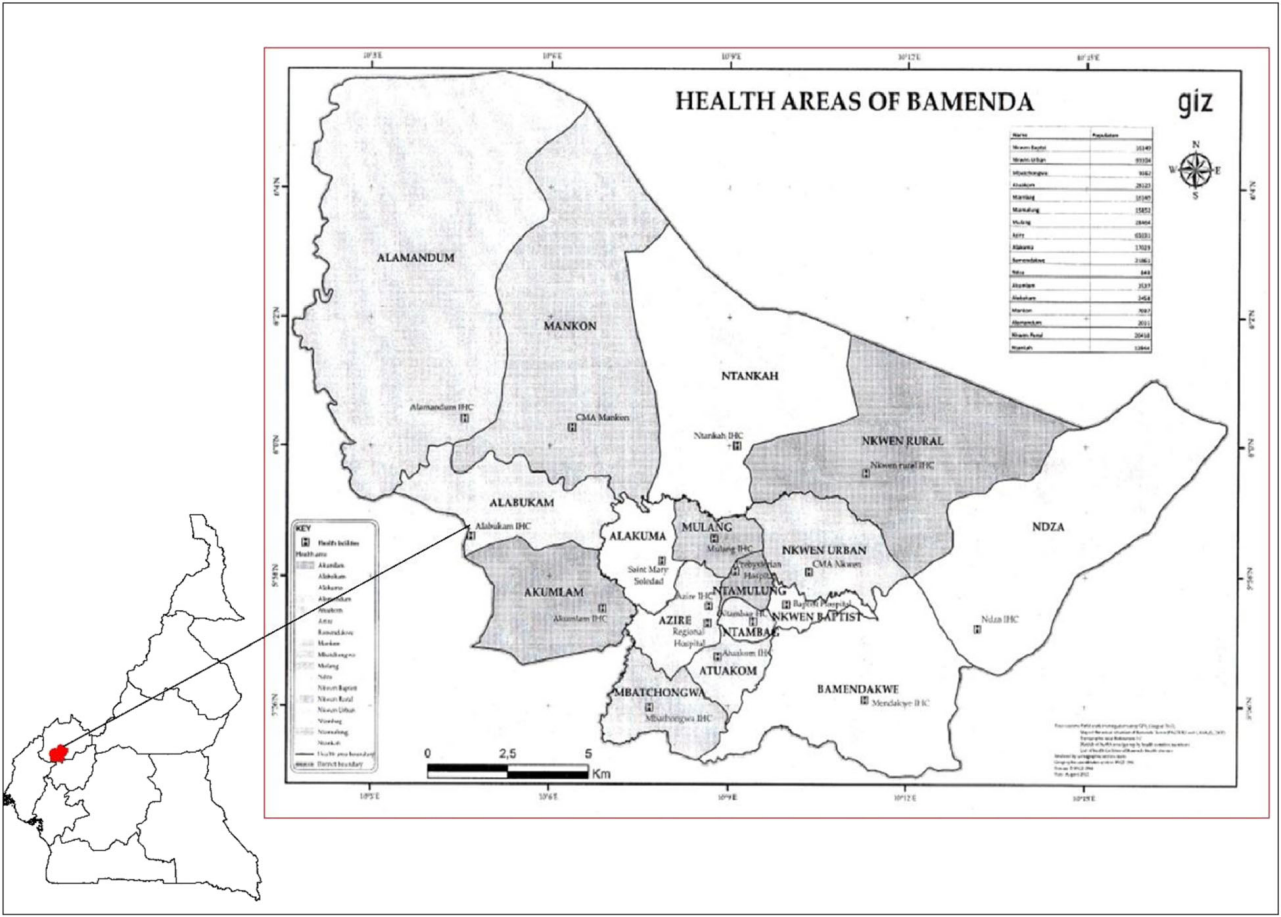
Map of Bamenda Health District

The study was conducted in the Bamenda Health District (BHD) within the High Western Plateau of Cameroon (Fig. 1). The weather is warm and wet most of the year. Bamenda doubles as the administrative headquarters of Mezam Division and for the North West region of Cameroon. It is a cosmopolitan city made up of three towns: Mankon, Nkwen and Bamendankwe and is inhabited by people originating from all over the country, and from neighbouring countries.

The BHD was selected for the study because it is a major urban centre in the country, with an important mix of people and cultures, and was covered by the 2011 mass-campaign of LLINs distribution. The BHD is composed of 17 health areas and has an estimated population of over 350,000 inhabitants. The population is mainly made up of people of the middle class with a majority of the inhabitants depending on small to big business ventures as a source of income.

### Study population

Seven of the 17 health areas in the BHD were randomly selected by simple draw. The randomly selected health areas were: Alakuma, Azire, Mankon, Nkwen Rural, Nkwen Urban, Ntambag and Ntamulung health areas. A sample size of 384 had been determined by the following formula [9]:

$$ \mathbf{N}=\frac{{\left(\mathbf{Z}\right)}^{\mathbf{2}}\mathbf{P}}{{\mathbf{D}}^{\mathbf{2}}}*\left(\mathbf{1}-\mathbf{P}\right) $$

Where, *N* = sample size Z^2^ = (1.96)^2^ for 95% confidence interval (that is α = 0.05, *P* = proportion of population owning at least a bednet (52%) D^2^ = maximum tolerable error for the prevalence estimate (0.05).

A total of 384 households were surveyed from these health areas. A household could include a single individual of either gender, or a compound with several people; only households where at least one person had spent the night prior to our visit in the house were surveyed.

### Study design and sampling strategy

This was a cross-sectional and community-based study conducted in the months of May and June 2014. A two-stage cluster sampling strategy was used. This entailed the random selection of seven health areas at the first stage and secondly, the random selection of clusters from these health areas. Each cluster was made up of 15 households, and a total of 25 clusters plus nine households were surveyed. In each household, one or both parents present were interviewed to obtain the information needed to fill the questionnaires.

### Sample collection

A semi-structured questionnaire (Additional file 1) was used to record data from all participating households after the consent form had been signed. The questionnaire was made up of six sections: demographic characteristics, socioeconomic status, ownership of LLINs/ITNs, knowledge of bednets, utilisation of bednets, and the household perceptions of LLINs/ITNs. The questionnaire was pretested in Buea city and after analysis, questions for which participants had difficulties understanding were rephrased. The socioeconomic status, knowledge of bednets and the environmental suitability of households for mosquito breeding were determined as shown on Table 1.

**Table 1:** Categorization of some predictive factors of bednet ownership

SN	Parameters	Categorized
1.	Socioeconomic/living standard = household assets owned amongst which are: TV, radio, refrigerator, vehicle, sanitary system, water availability, cooking fuel, electricity availability etc.	“Low” (have none of the indicators), “medium/middle” (have at least half of the mentioned indicators) and “high socioeconomic status” (has all of the indicators).
2.	Knowledge of malaria = knowledge on malaria transmission by mosquito vector and the proper use of bednets	“good” (has a good knowledge on malaria, it’s transmission and use of nets) and “poor” Knowledge (no or limited knowledge on malaria, it’s transmission and proper use of bednets
3.	The environment = swampy areas, water ponds or rivers/streams, presence of bush/forest and household waste.	“Less suitable” (no bushes, swampy areas, water ponds) or “very suitable” (the presence of bushes and dirty surroundings, rivers/streams for mosquito proliferation.

Dependent Variables: Two dependent variables were studied: bednet ownership defined as the household with at least one bednet, either used or not used; and bednet usage defined as sleeping under bednet the previous night.

### Statistical analyses

All data were entered and analysed using IBM-SPSS Statistics 20 for windows (IBM-SPSS Corp., Chicago USA). Descriptive statistics were done to characterise the demographic and intra-household characteristics and the bednet ownership and utilisation rates. The significance of difference in association between the predictive factors and bednet ownership and utilisation were explored using logistic regression analyses. The associated factors from the logistic regression analyses (significant level < 0.05) were used in multinomial logistic regression analysis to determine categorical differences in association. A difference giving a *p* value < 0.05 was considered statistically significant.

## Results

### Baseline demographic and intra-household data

From the 384 households surveyed, 1895 individuals were counted: 883 (46.6%) males and 1012 (53.4%) females. Out of the total number of individuals counted, 243 (12.8%) were children under 5 years and 13 (3.4%) pregnant women. Two hundred and seventy (70.3%) households were headed by a male and 114 (29.7%) by females. The mean age (± SD) of household heads was 40.05 ± 13.69 years (range 21–86). Ages were grouped into five categories: 21–30, 31–44, 41–58, 59–72 and 73–86.

Two hundred and thirty eight households (62%) were headed by married persons, 86 (22.4%) by singles, 47 (12.2%) by widowed heads and 13 households (3.4%) by divorced heads. Educational levels ranged from no formal education to tertiary education. Two hundred and one (52.3%) household heads had studied up to the primary educational level, followed by 120 (31.3%) with secondary education, 45 (11.7%) with tertiary education, and lastly those with no formal education in 18 households (4.7%). The occupational status of household heads was classified into six categories ranging from traders/self-employed (45.1%), salaried workers (27.6%) who were most represented and the unemployed (2.6%) and retirees (1.5%) were least represented.

Households had from 1 to 21 individuals (mean 4.93 ± 2.88). The household size was grouped into four categories: 189 (49.2%) with 1–4 people; 163 (42.4%) with 5–8 people, 24 (6.3%) with 9–12 people and 8 (2.1%) with 13 persons and above.

For the socioeconomic status, 184 (47.9%) households were in the middle socioeconomic class, 103 (26.8%) households in the high class and 97 (25.3%) households the low socioeconomic class. A good knowledge of bednets was found in 358 (93.2%) households and 26 (6.8%) households had a poor knowledge. Source of knowledge included from: health personnel (288; 80.7%), media sources (radio and television) (42; 11.8%) and from public sources (parents, relations, neighbours etc.) (27; 7.6%). Two hundred and ninety four (76.6%) households were located in place with few apparent suitable mosquito breeding grounds (larval developmental and adult resting sites) and 90 (23.4%) in areas with obvious suitable breeding sites for mosquitoes such as bushes and pools of standing water.

### Bednet ownership and utilisation

Two hundred and forty four of the 384 households owned at least a bednet, giving a bednet ownership frequency of 63.5%. Of the 1895 people covered in this study, 1036 were in households that owned at least a bednet (68.9%). A total of 470 bednets were found in all households, 91.9% of which were LLINs and 8.1% ITNs. From these figures, an ownership rate of 1.9 bednets per household and 2.2 persons per bednet was calculated in homes that owned nets. Bednet numbers ranged from 1 to 7 (2 ± 0.63) in households. Main sources of bednets were from the free mass distribution campaign (87.7%), antenatal clinic [ANC] (7%), gifts (3.3%) and purchased (2.1%).

Out of 244 households that owned at least a bednet, at least one person in 169 (69.3%) of them had slept under a net the previous night (Table 2). And out of 1306 individuals in households possessing at least a bednet, 616 (259 males and 357 females) individuals slept under a net the previous night 47.2%. Non-usage was attributed to negligence in putting nets down (29.3%); heat problems (26.7%); suffocation (10.7%); nets torn (8%); and other reasons (25.3%) including not hung, washed, expired, seasonal use, and not beneficial.

**Table 2:** Bednet utilization rates in different groups and pregnant women

Group	Number of persons in households that own nets	Number that slept under a bednet
Children < 5 years of age	243	153 (63%)
6–25 years of age	624	253 (40.5%)
26–49 years of age	350	174 (49.7%)
Above 50 years of age	79	30 (38%)
Pregnant women as a vulnerable group in the population		
Pregnant women	10	6 (60%)

Pearson *χ* ^2^ = 39.56, *p* = 0.009

### Bivariate logistic regression analysis of the association of predictive factors on bednet ownership and utilisation

#### Relationship between predictive factors and bednet ownership

**Fig. 2:**
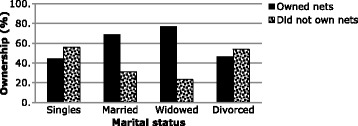
Relationship between marital status and bed net ownership

**Fig. 3:**
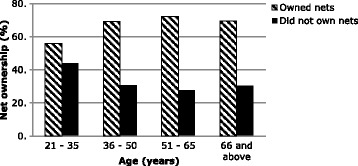
Relationship between Age of household head (categorised) and bed net ownership

**Fig. 4:**
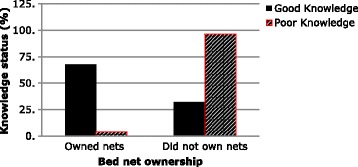
Relationship between Knowledge of bed nets and bed net ownership

Bednet ownership (Table 3) was associated to marital status of the household head (Fig. 2) where families with married heads significantly owned more nets than the other groups (*p* = 0.001). Age of the household head (Fig. 3) influenced bednet ownership where household heads aged between 51 and 65 years owned significantly more nets than the other age groups (*p* = 0.016). Knowledge on bednets (Fig. 4) whereby households where the head had a good knowledge of bednets owned nets more than those households with poor knowledge (*p* < 0.001).

**Table 3:** Factors associated to bednet ownership by bivariate analysis

Bednet ownership					
SN	Predictive factors	Significance (*p* value)	Odds ratio (OR)	95% Confidence interval (CI) for OR	
				Lower	Upper
1.	Marital status	0.001	0.64	0.49	0.84
2.	Knowledge	<0.001	52.8	7.07	394.67
3.	Age of household head	0.016	0.74	0.58	0.95
4.	Household size	0.002	0.59	0.43	0.83

#### Relationship between predictive factors and bednet utilisation

Bednet utilisation was associated to gender of household head whereby households with male heads owned nets more often than those with female heads (*p* = 0.048) (Table 4). Bivariate analysis showed that the level of education of the household head significantly (*p* = 0.029) influenced bednet use; however there was no significant difference using multivariate analysis.

**Table 4:** Factors associated with bednet utilisation by bivariate analysis

SN	Factors	*p* value	Odds ratio (OR)	95% Confidence interval (CI) for OR	
				Lower	Upper
1.	Gender	0.048	1.79	1.01	3.19
2.	Educational level	0.029	1.32	1.03	1.69
3.	Environmental suitability	0.035	2.18	1.06	4.49
4.	Bednet density	0.002	0.43	0.25	0.74
5.	Group categorisations	0.009	1.19	1.05	1.36

Households in suitable mosquito breeding sites had a higher bednet density (*p* = 0.002) and used nets more often than those in less suitable environments (*p* = 0.035).

For bednet usage in different age groups, both children less than 5 years and pregnant women used nets more than the other age groups (*p* = 0.009).

### Multinomial logistic regression analysis

#### Association between associated predictive factors and bednet ownership

Households with single heads (Table 5) were significantly less likely to own bednets compared to households with married heads (*p* < 0.001, OR = 0.35, 95% C.I. 0.21–0.59). Households with divorced and widowed heads were less and more likely respectively to possess bednets compared to married heads, but these differences were not significant (*p* = 0.098; OR = 0.39; 95% C.I. 0.13–1.19; *p* = 0.295; OR = 1.48; 95% C.I. 0.71–3.06 respectively).

**Table 5:** Multinomial logistic analysis showing the association between predictive factors and bednet ownership

DEPENDENT VARIABLE: Bednet OWNERSHIP						
SN	Variables		*p* value	Odds ratio	95% confidence interval for odds ratio	
1.	Marital status Reference category: Married.				Lower bound	Upper bound
	Singles	Ownership (yes)	<0.001	0.357	0.215	0.593
	Divorced	Ownership (yes)	0.098	0.387	0.126	1.191
	Widowed	Ownership (yes)	0.295	1.477	0.712	3.061
2.	Knowledge Reference category: Good knowledge					
	Poor knowledge	Ownership (yes)	<0.001	0.02	0.003	0.14
3.	Age of household head Reference category: 21–35 years of age					
	36–50	Ownership (yes)	0.018	1.77	1.10	2.85
	51–65	Ownership (yes)	0.035	2.05	1.05	3.99
	66 and above	Ownership (yes)	0.218	1.80	0.71	4.59
4.	Household size Reference category: 1–4 persons					
	5–8 persons	Ownership (yes)	0.001	2.08	1.33	3.24
	9–12 persons	Ownership (yes)	0.063	2.51	0.95	6.59
	13 and above persons	Ownership (yes)	0.139	5.01	0.59	42.42

Households where occupants had a poor knowledge of bednets were less likely to own nets compared to households where occupants had acceptable to good knowledge of bednets (*p* < 0.0001; OR = 0.02; 95% C.I. 0.003–0.14). Family heads aged between 36 and 50 years (*p* = 0.018); OR = 1.77; 95% C.I. 1.10–2.85) and 51–65 years (*p* = 0.035); OR = 2.05; 95% C.I. 1.05–3.99) significantly owned more bednets than those aged between 21 and 35 years of age. Households with 5–8 individuals were two times more likely to own bednets than households with 1–4 individuals (*p* = 0.001; OR = 2.08; 95% C.I. 1.33–3.24). Though households with 9–12 and greater than 13 individuals were 2.5 times and 5 times respectively more likely to own bednets than those with 1–4 individuals, these differences were not significant.

#### Association between associated predictive factors and bednet utilisation

Members of households with female heads were less likely to have slept (*P* < 0.001; OR = .56; 95% C.I. 0.31–0.99) under bednets when compared to occupants of homes headed by males (Table 6). Households situated in less suitable environment for malaria utilized less bed nets (*P* < 0.035; OR = .46; 95% C.I. 0.22–0.95). The level of education of household head did not influence the bed net utilization. Households having 3-4 nets were three times (OR = 3.1; 95% C.I. 1.47–6.55) more likely to have used at least a bednet the previous night compared to households with just one or two nets.. Children below 5 years of age were significantly more likely to have slept under bednets than people aged between 6 and 25 years (OR = 0.4; 95% C.I. 0.29–0.54; *p* < 0.0001), 26–49 years (OR = 0.58; 95% C.I. 0.42–0.81; *p* = 0.001) and those above 50 years of age (OR = 0.36; 95% C.I. 0.21–0.61; *p* < 0.0001).

**Table 6:** Multinomial logistic analysis showing the association between predictive factors and bednet utilisation

SN	DEPENDENT VARIABLE: Bednet UTILISATION					
	Variables		*p* value	Odds ratio (OR)	95% confidence interval (C.I.) for OR	
1.	Gender Reference category: Males				Lower bound	Upper bound
	Females	Utilised nets	0.048	0.56	0.31	0.99
2.	Environmental suitability Reference category: very suitable					
	Less suitable	Utilised nets	0.035	0.46	0.22	0.95
3.	Educational level Reference category: No formal education					
	Primary level	Utilised nets	0.842	0.87	0.22	3.39
	Secondary level	Utilised nets	0.778	0.82	0.2	3.33
	Tertiary level	Utilised nets	0.168	0.35	0.81	1.55
4.	Bednet density Reference category: 1–2 nets					
	3–4 nets	Utilised nets	0.003	3.1	1.47	6.55
	5 and more nets	Utilised nets	0.070	3.24	0.91	11.58
5.	Age groups Reference group: < 5 years					
	6–25 years	Utilised nets	< 0.0001	0.4	0.29	0.54
	26–49 years	Utilised nets	0.001	0.58	0.42	0.81
	> 50 years	Utilised nets	<0.0001	0.36	0.21	0.61
	Pregnant women	Utilised nets	0.849	0.88	0.24	3.21

## Discussion

This study aimed at determining the predictive factors of bednet ownership and utilisation in an urban area of the western highlands of Cameroon. This assessment took place 3 years post-first nationwide campaign of mass-distribution of free ITNs of 2011, to pave the way as the Ministry of Public Health, The National Malaria Control Programme and their partners prepared for the second campaign of free distribution. The frequency of bednet possession was 63.5% in households, and the frequency of utilisation was 69.3%, giving an overall utilisation frequency of 44% in the BHD.

Such an ownership rate 3 years post-distribution indicates that coverage might have been higher a couple of years back at the time of the mass-distribution campaign. This ownership frequency is higher than that obtained in others countries in the sub-region such as in Angola (52%), and may be accounted for by the fact that during the campaign of distribution in Cameroon, all households were included, unlike what happened in those countries where the distribution exercise was a targeted free distribution campaign [10, 11]. But at this point of our study, coverage was far below the WHO recommended level of 80% for acceptable protection [10]. Majority of the bednets were LLINs and a few were the old conventionally treated nets. The mass distribution campaign of 2011–2012 was the main source of the bednets. This is in line with reports from other countries such as Kenya [12]), Togo (95.2%) [13] and in Nigeria (81.5%) [14] and clearly demonstrates the pivotal role that mass-distribution campaigns play in ensuring that residents of endemic malaria areas own this key tool in the integrated fight against the disease.

The mean net density observed was approximately 2 bednets per household and 1 bednet for over two persons instead of two persons as is the target [1]. Most households had just 1 to 2 bednets (78.2%), while almost more than half of the homes surveyed (50.8%) had five and more people. Reasons advanced to justify this included: the small sizes of some households, other available nets were worn out, some were shared with those who did not have, nets were given to their children studying in other areas and also, the short supply of nets in some distribution centres.

With respect to household utilisation, 69% of households had at least used a net the previous night. Effective protection was however much lower when considering that only 47.2% of the total number of occupants of those houses slept under a net. This low usage by the population is confirmed by other findings [15, 16]. Findings from this study showed that negligence, heat, other reasons (not hung, washed, expired, dislike and house construction), discomfort/suffocation and torn nets were accountable for non-usage of bednets. This has been recorded from studies in Zaria, Northern Nigeria where bednet usage was also low, and non-usage of bednets was attributed to the factors similar to those outlined above (negligence, heat, other factors including nets not tied up, washed, expired, dislike and house under construction), discomfort/suffocation and torn nets [17].

Bednet use was highest in children under 5 years of age, and this is similar to results by Kroeger et al. [18] in South America. For the different age groups, findings of an investigation conducted in Uganda reported 63% of children less than 5 years having slept under mosquito nets throughout the night [19] which ties with findings from this study.

Bednet use in this study was next highest in pregnant women. This can be attributed to the emphasis that is laid on the benefits of sleeping under a bednet during pregnancy for the future mother and her baby through communication, especially community education when they attend antenatal clinics. During these visit in Cameroon, pregnant women also routinely receive free IPT-SP against malaria every 3 months, and those that do not have an ITN receive one. In addition, the aforementioned vulnerable groups (*viz*. children below five and pregnant women) were prioritised for use of bednets by members of their family in case of shortage of nets available to the household as had been observed previously [6, 8, 20]. In the other age groups, those aged 26–49 year slept more often under nets than school-aged children, adolescents and young adults (6–25 years) and those greater than 50 years of age. This is because most of those aged between 26 and 49 years are parents who tend to sleep under nets with their babies and younger children (this category also includes most pregnant women). But school-aged persons tend to sleep in separate beds or rooms without much control on proper bednet utilisation or due to bednet shortages [3]. Those aged more than 50 years preferred that younger children sleep under the nets rather than themselves especially with the net shortage experienced.

There was no impact of household size on bednet utilisation. However, studies carried out in Burkina Faso and South West Ethiopia respectively showed that the use of bednets was significantly lower in larger households than in smaller ones [21, 22]. Sleeping arrangements, bednet density and limited parental control can explain such results.

Associations were obtained between bednet ownership and marital status, age of household head, knowledge about bednets and household size which was in line with a similar study conducted in Kenya by Ng’ang’a et al. [23]. A positive association was obtained between knowledge on bednets and bednet ownership implying that the more knowledgeable a household was, the more likely they were to seek and own bednet(s). This observation is in concordance with findings by Graves et al. [8] in Ethiopia. Findings in this study and another one in Kenya [24] showed no association between socioeconomic status and bednet ownership and utilisation, because free mass-distribution campaigns in both studies decreased the socioeconomic inequity in bednet ownership. This is in contrast to findings of a study in Madagascar [25] where after free distribution, net ownership appeared influenced by the socioeconomic status with the least poor owning more bednets than the poorest communities. Knowledge on association between bednet ownership and some predictive factors is critical to improve on the success of the intervention strategy, especially as the country is preparing for another major mass-campaign of free distribution.

Household headed by married and widowed persons were more likely to own bednets than households headed by singles. This has been attributed to the much better decision making power in parented households and also their responsibility in protecting their offspring against disease [26]. Households with 5–12 individuals were more likely to own bednets than households with 1–4 individuals. This has previously been observed in a rural setting in the Democratic Republic of Congo. There, bednet ownership was positively correlated with the household density [27]. This was because these households were mostly headed by married or widowed heads that were more alert in decision making, and positively influenced acquisition of bednets to protect their families against malaria. Majority of households with 1–4 individuals were mostly made up of students and singles. As the household size increased above 13 persons, there was no significant difference in bednet ownership when compared to households with 1–4 individuals. This can be attributed to the limited control over such large numbers. Households heads aged between 36 and 65 years were more likely to own bednets in their households than in households with heads less than 35 years of age attributed to the fact that they are more decisive, knowledgeable, and more responsible than the younger heads [28]. Thus they tend to own nets more often than the younger heads that were either singles, students or younger adults.

In this study, there were associations between bednet utilisation and gender (with male headed households more likely to have used nets than female headed households) and the educational level of the household head, although when comparing the different levels of education no significant difference was obtained. It has been explained in the Cross River state of Nigeria [29] that educated parents may be better able to appreciate the importance of treated nets in malaria prevention and to understand the information included in the public awareness campaigns which will eventually influence bednet usage by their families. Such associations have also been shown by in Kinshasa-DRC by Pettifor et al. [30] and Ndjinga and Minakawa [31] and in Mfou-Cameroon by Tchinda et al. [3].

Environmental factors, bednet density and usage between different age groups were also significantly associated to bednet utilisation. Knowledge was not associated to bednet utilisation in this study but was otherwise shown in results obtained in the Mfou-Cameroon [3]. This was so because of negligence and the environmental conditions of heated nights.

## Conclusion

This study is significantly contributing to information on bednet ownership and usage in the BHD of Cameroon. Bednet ownership and utilisation rates remain below the WHO recommended standards of 80%. The factors of marital status and age of the household head, knowledge of bednets and the household size are relevant as they have both positive and negative impacts on bednet ownership. While the gender and educational level of the household head, bednet density in households, the environmental suitability and usage by different age groups are also relevant factors that have impacts on bednet utilisation in households. Bednets being a core vector control strategy, these data is important in understanding bednet coverage dynamics, which could facilitate decision making in order to improve bednet ownership and utilisation in the fight against malaria.

## Additional file

Additional file 1: Cluster and household number. (DOCX 498 kb)
