# Supplementary material for: Determination of the predictive factors of long-lasting insecticide-treated net ownership and utilisation in the Bamenda Health District of Cameroon
Source: BMC Public Health. 2017 Mar 16;17:263. doi: 10.1186/s12889-017-4155-5 (PMC5356302; doi:10.1186/s12889-017-4155-5)
Supplement: Additional file 1: — Cluster and household number. (DOCX 498 kb) [file 12889_2017_4155_MOESM1_ESM.docx]

**Additional file 1**

**CLUSTER AND HOUSEHOLD NUMBER**

Health Area ……………………. Cluster number ……………. Household number …………

Sex:

Age/years…………... Occupation ………………………. Level of Education …………...

Status: Single Married Divorced

| 1. **Bed net Ownership questions** | | | | | | | | | | | | |
| --- | --- | --- | --- | --- | --- | --- | --- | --- | --- | --- | --- | --- |
| **1.** | Ownership | Yes | | No | | |  | | | | | |
| **2.** | How was it obtained? | | Mass distribution | | | | | ANC | | | Bought | Gift |
| **3.** | Is a LLIN (1) or ITN (2)? | | 1 | | | 2 | | |  | | | |
| **4.** | How many does the HH possess? | | | | | | | | | | | |
| **5.** | Are there any pregnant women? | | | | Yes  No. ---- | | | | | No | |  |
|  | | | | | | | | | | | | |
| **b.** **UTILISATION** | | | | | | | | | | | | |

Household size ……………

| **6.** | Last night, how many sleeping places were there? | | | | | | | | | | | | | | | | | | | | | | No. -------------- | | | | | | | | | | | | | | | | | | | | | | | | | | | | |
| --- | --- | --- | --- | --- | --- | --- | --- | --- | --- | --- | --- | --- | --- | --- | --- | --- | --- | --- | --- | --- | --- | --- | --- | --- | --- | --- | --- | --- | --- | --- | --- | --- | --- | --- | --- | --- | --- | --- | --- | --- | --- | --- | --- | --- | --- | --- | --- | --- | --- | --- | --- |
| **7.** | How many people of all ages slept in the house the previous night? | | | | | | | | | | | | | | | | | | | | | | | | | | | | | | | | | | | | | | | | | | | | | | | | | | |
| **8.** | Did anyone sleep under this net last night? | | | | | | | | | | | | Yes | | | | | | No | | | | | | Not sure | | | | | | | | | | | | | | | | | | | | | | | | | | |
| SN | | | Code | | Age | Gender | | | Level of education | | | | | | | Slept under LLIN the previous night | | | | | | | | | | | | | | | |  | | | | | | | | | | | | | | | | | | | |
| 1. | | |  | |  |  | | |  | | | | | | |  | | | | | | | | | | | | | | | |  | | | | | | | | | | | | | | | | | | | |
| 2. | | |  | |  |  | | |  | | | | | | |  | | | | | | | | | | | | | | | |  | | | | | | | | | | | | | | | | | | | |
| 3. | | |  | |  |  | | |  | | | | | | |  | | | | | | | | | | | | | | | |  | | | | | | | | | | | | | | | | | | | |
| 4. | | |  | |  |  | | |  | | | | | | |  | | | | | | | | | | | | | | | |  | | | | | | | | | | | | | | | | | | | |
| 5. | | |  | |  |  | | |  | | | | | | |  | | | | | | | | | | | | | | | |  | | | | | | | | | | | | | | | | | | | |
| 6. | | |  | |  |  | | |  | | | | | | |  | | | | | | | | | | | | | | | |  | | | | | | | | | | | | | | | | | | | |
| 7. | | |  | |  |  | | |  | | | | | | |  | | | | | | | | | | | | | | | |  | | | | | | | | | | | | | | | | | | | |
| **c.** **KNOWLEDGE ON BED NETS** | | | | | | | | | | | | | | | | | | | | | | | | | | | | | | | | | | | | | | | | | | | | | | | | | | | |
| **11.** | What do you know about the proper use of bed nets? | | | | | | | | | | | | | | | | | | | | | | | | | | | | | Knows | | | | | Does not know | | | | | | | | | | | |  | | | | |
| **12.** | What is your main source of information about bed net use? | | | | | | | | | | | | | | | | | | | | | | | | | | | | | | | | | | | | | | | | | | | | | | | | | | |
| **13.** | Has anyone visited this HH in the last 6months to talk about malaria or bed nets? | | | | | | | | | | | | | | | | | | | | | | | | | | | | | Yes | | | | | No | | | | | | | Do not know | | | | | | | | | |
| **14.** | Has any HH member visited a health facility where there were talks on malaria or bed nets in the last 6months? | | | | | | | | | | | | | | | | | | | | | | | | | | | | | | | | | | | | | | Yes | | | | No | | | Do not know | | | | | |
| **15**. | Has any HH member heard or seen any media broadcast about bed nets in the last 6months? | | | | | | | | | | | | | | | | | | | | | | | | | | | | | | | | | Yes | | | | No | | | | | | Do not know | | | | | | | |
| **16**. | What is the main method used in your HH to prevent malaria? | | | | | | | | | LLIN/ITN | | | Mosquito repellent | | | | | | | | | Preventive treatment | | | | | | Indoor residual spraying(IRS) | | | | | | | | | Environmental hygiene | | | | | | | | | | | | Others | | |
|  | | | | | | | | | | | | | | | | | | | | | | | | | | | | | | | | | | | | | | | | | | | | | | | | | | | |
| **d. HOUSEHOLD SOCIOECONOMIC STATUES** | | | | | | | | | | | | | | | | | | | | | | | | | | | | | | | | | | | | | | | | | | | | | | | | | | | |
| **17**. | Does your HH have electricity? | | | | | | | | | | | | | Yes | | | | | | No | | | | | | | | | | | | | | | | | | | | | | | | | | | | | | | |
| Radio | | Television | | | Refrigerator | | | Electric iron | | | | Motorcycle | | | | | | | | | Car or truck | | | | | | | | Cattle, sheep, goat | | | | | | | | | | | Domestic worker | | | | | | | | | | | |
| **18.** | What is the principal HH source of drinking water | | | | | | | | | | | Tap water into residence | | | | | | | | | | | | | | Protected well in residence | | | | | | | | | | | | | | Unprotected well in residence | | | | | | | | | | | |
| Public tap | | | | Unprotected public well | | | Protected public well | | | | | | | | | | Bottled water | | | | | | | | | Rain water | | | | | | | Surface water (streams, rivers etc.) | | | | | | | | | | | | | | | | | Spring | |
|  | | | |  | | |  | | | | | | | | | |  | | | | | | | | |  | | | | | | |  | | | | | | | | | | | | | | | | |  | |
| **19.** | What is the principal type of toilet/sanitary facility used by the HH? | | | | | | | | | | Own flush toilet | | | | | | | Shared flush toilet | | | | | | | | | Own pit latrine | | | | | | | | | Shared pit latrine | | | | | | | | | Bush or field | | | | | | Others |
| **20.** | What is the principal type of cooking fuel in your HH? | | | | | | | | | | | | | | Wood or dung | | | | | | | | | Kerosene | | | | | | | Charcoal | | | | | Electricity | | | | | Gas | | | | Solar | | | Others (specify) | | | |
| **e. HOUSEHOLD VIEWS** | | | | | | | | | | | | | | | | | | | | | | | | | | | | | | | | | | | | | | | | | | | | | | | | | | | |
| **21.** | How do you use your bed nets? | | | | | | | | | | | | | | | | | | | | | | | | | | | | | | | | | | | | | | | | | | | | | | | | | | |
| **22.** | From your point of view, has the mass distribution benefitted your HH by cutting down malaria frequency? | | | | | | | | | | | | | | | | | | | | | | | | | | | | | | | | | | | | | | | | | | | | | | | | | | |
| **FOR NON USERS** | | | | | | | | | | | | | | | | | | | | | | | | | | | | | | | | | | | | | | | | | | | | | | | | | | | |
| **23.** | Why don’t you sleep under a bed net? | | | | | | | | | | | | | | | | | | | | | | | | | | | | | | | | | | | | | | | | | | | | | | | | | | |
| **27.** | What do you think can be done to boost use among non-users? | | | | | | | | | | | | | | | | | | | | | | | | | | | | | | | | | | | | | | | | | | | | | | | | | | |
